# Surveillance of infections of surgical sites and lower respiratory tracts should be combined: experiences from the German surveillance module for operated patients (OP-KISS), 2018 to 2022

**DOI:** 10.2807/1560-7917.ES.2024.29.11.2300416

**Published:** 2024-03-14

**Authors:** Seven Johannes Sam Aghdassi, Selin Saydan, Michael Behnke, Jörg Clausmeyer, Petra Gastmeier, Christine Geffers

**Affiliations:** 1 Charité – Universitätsmedizin Berlin, corporate member of Freie Universität Berlin and Humboldt-Universität zu Berlin, Institute of Hygiene and Environmental Medicine, Berlin, Germany; 2 National Reference Centre for Surveillance of Nosocomial Infections, Berlin, Germany; 3 Berlin Institute of Health at Charité – Universitätsmedizin Berlin, BIH Biomedical Innovation Academy, BIH Charité Digital Clinician Scientist Program, Berlin, Germany

**Keywords:** surveillance, lower respiratory tract infection, pneumonia, surgical site infection, surgical patients, Infection control

## Abstract

**Background:**

Surveillance of lower respiratory tract infections (LRTI) of operated patients conventionally focuses on intubated patients in intensive care units (ICU). Post-operative immobilisation increases the risk of LRTI not associated with ventilators. Operated patients, however, have thus far not been a primary target for LRTI surveillance.

**Aim:**

We aimed to describe the applied LRTI surveillance method in the German surveillance module for operated patients (OP-KISS) and to report data between 2018 and 2022.

**Methods:**

Surveillance of LRTI can be performed voluntarily in addition to surgical site infection (SSI) surveillance in OP-KISS. We calculated LRTI rates per 100 operations for all procedures combined, as well as for individual surgical groups and procedures. Additionally, a combined post-operative infection rate (SSI and LRTI) was calculated.

**Results:**

Surveillance of LRTI was performed in 4% of all participating OP-KISS departments and for 2% (23,239 of 1,332,438) of all procedures in the OP-KISS database. The pooled LRTI rate was 0.9 per 100 operations, with marked differences between different types of surgery (3.6 for lobectomies, 0.1 for traumatology and orthopaedics). The share of LRTI among all post-operative infections was highly variable. For lobectomies, the LRTI rate was higher than the SSI rate (3.6 vs 1.5 per 100 operations).

**Conclusion:**

Surveillance of post-operative LRTI is not yet widely adopted by German hospitals. Based on the data in this study, lobectomies represent a prime target for post-operative LRTI surveillance.

**Key public health message**

**What did you want to address in this study?**

Due to post-operative immobilisation, operated patients are at a high risk of lower respiratory tract infections. However, the problem is somewhat under-investigated. Here, we share experiences and data from a surveillance method for lower respiratory tract infections in operated patients in Germany.

**What have we learnt from this study?**

Only a small fraction (4%) of the surgical departments participating in the German surveillance module for operated patients collected data on lower respiratory tract infections. Still, our study showed that post-operative respiratory infections occurred after around one percent of operations. Following lung removal procedures, the rate was higher (4%).

**What are the implications of your findings for public health?**

The study shows that surveillance of post-operative respiratory infections is feasible outside the scope of intubated patients in intensive care units, and it can detect a relevant number of infections. Insights from this study could help with wider implementation of lower respiratory tract infection surveillance.

## Introduction

Lower respiratory tract infections (LRTI) are among the most frequently occurring healthcare-associated infections (HAI) in European hospitals [1-3]. These infections affect patients in intensive care units (ICU) particularly often [4]. The majority of LRTI in ICU are associated with mechanical ventilation, which represents a major risk factor for LRTI occurrence [5-7]. Accordingly, infection prevention and control (IPC) recommendations for LRTI prevention predominately focus on intubated patients [8] and most LRTI surveillance activities focus on patients in ICU [9,10]. However, given that most hospitalised patients are not treated in ICU and are not intubated, this raises the question, whether the risk of LRTI in non-ventilated patients is adequately appreciated by current surveillance standards.

As demonstrated by the European Centre for Disease Prevention and Control (ECDC) point prevalence survey (PPS) on HAI and antimicrobial use conducted in 2011 and 2012 in various European countries, LRTI are a common phenomenon outside of ICU, accounting for almost one fifth of all non-ICU HAI [4]. Moreover, most of all healthcare-associated pneumonia cases documented in the German PPS 2016 were not ventilator-associated [11]. Aggregated European data from the ECDC PPS 2011, revealed similar results with only around one third of all documented pneumonia cases being ventilator-associated [4]. The LRTI complicate the post-operative process and are significantly associated with a prolonged post-operative hospital stay and increased mortality [12,13], clearly demonstrating the need to concentrate IPC efforts on preventing LRTI in operated patients.

Surveillance of LRTI on ICU can be standardised rather effectively, given the high prevalence of intubated patients and the comparatively high similarity regarding severity of disease. Patients outside of ICU are typically more heterogeneous and risk profiles for HAI differ substantially by specialty, rendering the development of a suitable surveillance method for LRTI more difficult. Many operated patients, however, share post-operative immobilisation as a common feature that increases the risk of LRTI. Consequently, the German surveillance module for operated patients (OP) as part of the German national nosocomial infection system (Krankenhaus-Infektions-Surveillance-System (KISS)), which primarily focuses on surgical site infections (SSI), also enables participating departments to monitor operated patients for the occurrence of LRTI [14].

Given the scarcity of LRTI surveillance in non-ICU patients outside of prevalence surveys, we aim to describe the applied LRTI surveillance methodology in OP-KISS and report results from a 5-year period between 2018 and 2022.

## Methods

The data used for this study were from the module OP-KISS. The authors provide additional information about KISS in the Supplementary file Additional File 1. The module OP-KISS focuses on hospital patients undergoing specific types of surgery, so-called indicator procedures [15]. An indicator procedure is defined as a set of procedure codes describing similar kinds of operations. Indicator procedures are grouped into eight distinct surgical groups. Box lists the surgical groups, as well as the respective individual indicator procedures for the unspecific surgical group general surgery.

Box **Surgical groups of indicator procedures in the surveillance module for operated patients in the German national nosocomial infection system**

**General surgery:**

- Hernia repair (inguinal)
- Lobectomy
- Thyroid surgery
- Parotidectomy
- Neck dissection
**Abdominal surgery**
**Traumatology and orthopaedics**
**Urology**
**Gynaecology**
**Cardiac surgery**
**Vascular surgery**
**Neurosurgery**

The authors provide selected additional methodological details of OP-KISS in the Supplementary file Additional File 1. Since 2012, surveillance of LRTI can be performed voluntarily in addition to SSI surveillance for any of the selected indicator procedures. Case definitions for LRTI in OP-KISS encompass pneumonia and bronchitis and are largely in alignment with ECDC HAI case definitions [16]. The authors present key criteria for the LRTI case definitions in the Supplementary file Additional File 1.

### Data source

Data from a 5-year period (2018–2022) were extracted from the OP-KISS database in May 2023. Only data from German surgical departments were included. The decision to select data from 2018 onwards, was because the importance of LRTI surveillance in OP-KISS was explicitly addressed during the 2017 meeting of KISS participants. All indicator procedures were considered. The OP-KISS methodology requires participating departments actively search for and document infections for all included procedures (active surveillance). Datasets, for which participants indicated that active surveillance was not carried out, were excluded. Surgeries with missing data were excluded as well. Retrospective data entry into the OP-KISS database by participants is possible at any time, which explains potential differences to OP-KISS data published elsewhere (e.g. reference data).

### Data analysis

For analysis of LRTI data, indicator procedures were merged into the above-mentioned eight surgical groups. The number of departments conducting LRTI surveillance and their share among all participating departments was calculated. Participation was defined as having at least one procedure with active surveillance in the OP-KISS database. Similarly, the number and proportion of procedures with LRTI surveillance was determined. Rates of LRTI per 100 operations were calculated for all procedures combined, as well as for individual surgical groups. For surgical groups with LRTI data from less than five different surgical departments, or less than 1,000 procedures with LRTI surveillance documented in total, no separate rates were calculated. Additionally, combined infection rates (LRTI and SSI) were estimated by adding up the rate of LRTI per 100 operations and SSI per 100 operations. All SSI, also infections that occurred in procedures with SSI but without LRTI surveillance, were considered for this purpose.

## Results

Altogether, 62 departments conducted LRTI surveillance in OP-KISS at least temporarily during the 5-year study period. Compared with 1,589 departments that performed SSI surveillance during this period, this corresponded to 4% of all participating departments. Between 2018 and 2022, a total of 1,332,438 procedures fulfilling inclusion criteria were entered into the OP-KISS database. For 23,239 (2%) of these, active surveillance of LRTI was performed. The pooled LRTI rate for the entire study period across all surgical disciplines was 0.9 per 100 operations. Figure depicts, for all surgical groups combined, the percentage of departments conducting LRTI surveillance as a proportion of all participating departments and the share of procedures with LRTI surveillance among all procedures over time.

**Figure:**
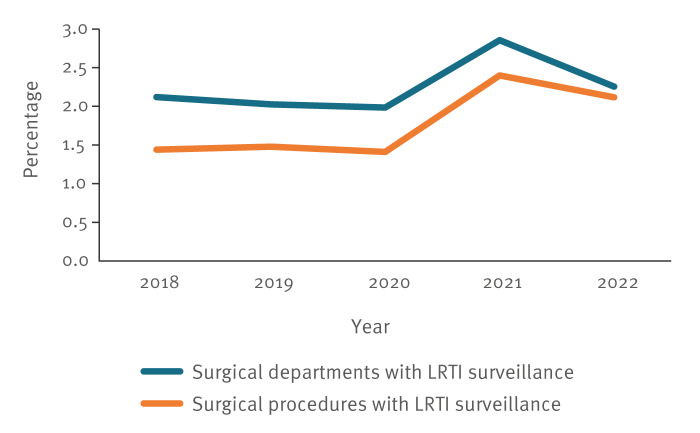
Proportion of departments and procedures with surveillance of lower respiratory tract infections among all participating surgical departments (n = 1,589) and all surgical procedures (n = 1,332,438), Germany, 2018–2022

LRTI: lower respiratory tract infections.

The number of participating surgical departments with lower respiratory tract infection surveillance in the different years were: 25 in 2018, 25 in 2019, 24 in 2020, 33 in 2021 and 26 in 2022.

The number of surgical procedures with lower respiratory tract infection surveillance in the different years were: 1,179 in 2018, 1,233 in 2019, 1,209 in 2020, 1,155 in 2021 and 1,152 in 2022.

The proportion of departments conducting LRTI surveillance remained at a stable but low level throughout the study period (range: 2.0–2.9). Similarly, the proportion of procedures with LRTI surveillance stagnated at a low level (range: 1.4–2.4). A slight increase in the year 2021 was noted.

For only three of eight surgical groups (general surgery, abdominal surgery, traumatology and orthopaedics) at least 1,000 procedures with LRTI surveillance from a minimum of five different surgical departments were documented. Of 7,020 procedures with LRTI surveillance in general surgery, 4,000 (57%) were lobectomies, 2,684 (38%) were thyroid surgeries, 289 (4%) were inguinal hernia repairs and 47 (1%) were parotidectomies. No neck dissections with LRTI surveillance were documented. All LRTI documented in the group general surgery occurred after lobectomies. Accordingly, data will be presented in the following for lobectomies, rather than general surgery as a whole.

Table 1 summarises the number of departments conducting LRTI surveillance and the number of procedures with LRTI surveillance, for all procedure types combined, as well as separately for lobectomies, abdominal surgery and traumatology and orthopaedics. While Table 1 contains aggregated data of 5 years, the authors present data per year in Supplementary Table S1 of the Supplementary file Additional File 2.

**Table 1:** Surveillance of lower respiratory tract infection in surgical departments participating in the national nosocomial infection system (OP-KISS), Germany, 2018–2022

Procedure type	Departments				Procedures		
	LRTI	SSI		%	LRTI	SSI	%
All procedures	62	1,589		3.9	23,239	1,332,438	1.7
Lobectomy	12	39		30.8	4,000	10,936	36.6
Abdominal surgery	14	403	3.5		1,672	186,483	0.9
Traumatology and orthopaedics	24	721	3.3		8,865	666,198	1.3

LRTI: lower respiratory tract infections; OP-KISS: the German surveillance module for operated patients (OP) as part of the German national nosocomial infection system (Krankenhaus-Infektions-Surveillance-System (KISS)); SSI: surgical site infections.

Altogether, traumatology and orthopaedics had the highest number of procedures with LRTI surveillance. The relative proportion of departments and procedures with LRTI surveillance, was considerably higher for lobectomies than for the other surgery types.

Table 2 shows the number of procedures with active SSI and LRTI surveillance, the number of SSI and LRTI, as well as the rates for SSI and LRTI per 100 operations for lobectomies, abdominal surgery and traumatology and orthopaedics. Additionally, Table 2 displays the combined post-operative infection rate (SSI and LRTI per 100 operations). While Table 2 contains aggregated data of 5 years, the authors present data per year in Supplementary Table S2 of Supplementary file Additional File 2.

**Table 2:** Surgical site infections, lower respiratory tract infections and combined post-operative infections per 100 operations, Germany, 2018–2022

Procedure type	LRTI surveillance			SSI surveillance			Combined post-operative infection rate^a^
	Number of infections	Number of procedures	Rate^a^	Number of infections	Number of procedures	Rate^a^	
Lobectomy	144	4,000	3.6	162	10,936	1.5	5.1
Abdominal surgery	6	1,672	0.4	6,227	186,483	3.3	3.7
Traumatology and orthopaedics	6	8,865	0.1	5,759	666,198	0.9	0.9

LRTI: lower respiratory tract infections; SSI: surgical site infections.

^a^ Per 100 operations.

The LRTI rate was markedly higher for lobectomies (3.6 per 100 operations) than for abdominal surgery (0.4 per 100 operations) and for traumatology and orthopaedics (0.1 per 100 operations). For lobectomies, 71% of all documented post-operative infections per 100 operations were LRTI. Conversely, the share of LRTI among post-operative infections for abdominal surgery (10%) and traumatology and orthopaedics (7%) was considerably lower.

Rates of LRTI per 100 lobectomies were higher in 2021 and 2022 (4.8 in 2021, 6.6 in 2022) than in 2018–2020 (2.6 in 2018, 3.0 in 2019, 2.5 in 2020). For other procedure types, LRTI rates over time showed only little fluctuation.

## Discussion

The findings of this study demonstrate a rather low uptake of LRTI surveillance by OP-KISS participants, which resulted in a much smaller database than for SSI surveillance. Consequently, the observed LRTI rates must be interpreted with caution. Our findings indicate that the frequency of LRTI vary by type of procedure. After lobectomies, LRTI occurred more frequently than SSI, illustrating the usefulness of LRTI surveillance after this surgery.

The comparatively limited engagement by departments with LRTI surveillance is in stark contrast to the otherwise high degree of implementation of HAI surveillance in Germany [17,18]. Although organised in the same KISS module (OP-KISS), only 4% of departments performing SSI surveillance also monitored patients for LRTI. Unlike for SSI, conducting surveillance of post-operative LRTI is not mandated by law in Germany [19], which likely contributed to surgical departments only placing subordinate importance on post-operative LRTI surveillance. Moreover, LRTI surveillance was only possible for OP-KISS participants under the condition that they also perform SSI surveillance for the respective indicator procedures. However, SSI surveillance represents a laborious task and might already consume a notable portion of available IPC resources at many hospitals [20,21]. It is therefore likely that many potentially interested departments decided to not conduct LRTI surveillance in OP-KISS, not because of lack of interest but because of limited IPC resources. Three of the five considered years were heavily influenced by the coronavirus disease 2019 (COVID-19) pandemic. This could have affected the willingness to conduct surveillance in both directions. As a respiratory disease, COVID-19 might have positively influenced awareness and readiness to perform surveillance for respiratory infections, including post-operative LRTI. On the other hand, healthcare resources were substantially stretched, which in turn might have reduced time and willingness to perform additional surveillance. Of note, LRTI rates after lobectomy were considerably higher in 2022 and 2021 than in previous years. However, given the limited annual number of procedures, interpretations of trends over time or concerning a connection with the COVID-19 pandemic, have to be made with caution and remain speculative at this point.

We observed a high LRTI rate following lobectomies. This is in alignment with other studies revealing high LRTI rates after thoracic surgery [12]. This finding confirms rather intuitive assumptions, as lobectomies reduce lung capacity and frequently lead to pain-induced shallow breathing, which are known risk factors for LRTI occurrence. Based on this observation, thoracic surgery should be a primary target for LRTI surveillance and prevention strategies. It is striking that in our dataset, lobectomies were not only the surgery type with the highest LRTI rate, but also the one with the highest percentage of departments performing LRTI surveillance. It is therefore conceivable that the rate of LRTI following lobectomies was in a more realistic range than for other surgery types and might more accurately reflect the actual burden of post-operative LRTI. To determine additional procedure types, for which post-operative LRTI surveillance is particularly important, additional studies and more robust data will be required.

Comparisons with other studies on post-operative LRTI have to be made with caution, as the share of surgical groups differs between the respective datasets. Metersky et al. performed a retrospective cohort study with data from over 58,000 patients that underwent major surgical procedures between 2009 and 2019 [22]. The data were extracted from the Medicare Patient Safety Monitoring System, an extensive adverse-event database in the United States. The authors found a pooled post-operative pneumonia rate of 1.9 per 100 procedures during 2009–2011 that decreased to 1.3 during 2017–2019, which is slightly higher than the rate observed in our study. Caparelli et al. examined the occurrence of pneumonia in non-cardiac surgical patients at a 209-bed community hospital in Ohio between 2014 and 2016 and found an incidence of 0.8 per 100 procedures before initiation of a targeted LRTI prevention programme [23]. Chughtai et al. analysed data from two American databases, the American College of Surgeons National Surgical Quality Improvement Programme and the Nationwide Inpatient Sample, to determine the rate of post-operative pneumonia after different surgical procedures [24]. An overall incidence of around 1.0 per 100 operations was found for both databases, which is comparable to the results of our study. Despite pronounced differences in the procedure types included, which as demonstrated by our results heavily influences the frequency of post-operative LRTI, there appears to be a certain degree of congruence between the results from other studies and our data.

Several limitations have to be acknowledged when interpreting the study findings. Firstly, LRTI surveillance was performed for only a small portion of procedures included in OP-KISS, thus reducing the amount of available data. Secondly, the number of procedures with LRTI surveillance varied greatly between the different surgical groups, complicating comparisons between the groups. Thirdly, the estimated combined post-operative infection rate was a crude addition of SSI and LRTI rates per 100 operations, with the denominator for LRTI rates being substantially smaller. Fourthly, since surveillance was terminated in case of re-operation, LRTI occurring after re-operation were not counted, resulting in a potential underestimation of LRTI rates. Fifthly, surveillance of LRTI could only be performed in addition to SSI surveillance by activating the LRTI component of the respective indicator procedure in in the KISS online portal. The low LRTI rates after trauma and orthopaedic procedures could also be partly attributable to the possible phenomenon that in some cases, while SSI surveillance was continued, LRTI surveillance was stopped without deactivating the LRTI component in the KISS online portal. This would result in ongoing documentation of denominator data (operations) without numerator data (LRTI), thus artificially lowering LRTI rates. Sixthly, concerning lobectomies, the distinction between SSI and LRTI was more difficult than for the other procedure types. It is conceivable that some SSI involving the parenchyma of the lung were misclassified as LRTI. However, to minimise this potential, participants were instructed to record infections, such as an abscess or empyema in the lung or thorax, as SSI rather than LRTI. The National Reference Centre for HAI surveillance also specified that in situations, where a lung abscess or pleural empyema was present along with pneumonia, SSI rather than LRTI should be recorded. Nonetheless, with regard to the increased LRTI rates after lobectomy, the possibility that SSI were incorrectly classified as LRTI must be considered. However, any distortion due to this phenomenon would not affect the combined post-operative infection rate. Lastly, data were collected by local staff with presumable differences in sensitivity and specificity regarding HAI case finding. However, to counteract this potential distortion, all departments participating in OP-KISS must undergo specific training by the National Reference Centre for HAI surveillance.

## Conclusions

Surveillance of post-operative LRTI is not yet implemented widely in German hospitals. The feasibility of monitoring LRTI for operated patients outside of ICU, however, was demonstrated by over 60 departments performing LRTI surveillance in OP-KISS during the study period. Based on the data in this study, lobectomies represent a primary target for post-operative LRTI surveillance. Further studies will be required to identify additional procedure types, for which post-operative LRTI surveillance is particularly useful. The experiences described in this article are intended to motivate other institutions to develop and implement methods for LRTI surveillance for operated patients.

## Supplementary Data

547000 Supplementary Material 1

## Supplementary Data

546000 Supplementary Material 2
