# Supplementary material for: Surveillance of infections of surgical sites and lower respiratory tracts should be combined: experiences from the German surveillance module for operated patients (OP-KISS), 2018 to 2022
Source: Euro Surveill. 2024 Mar 14;29(11):2300416. doi: 10.2807/1560-7917.ES.2024.29.11.2300416 (PMC10941308; doi:10.2807/1560-7917.ES.2024.29.11.2300416)
Supplement: Supplementary Material 1 [file 23-00416_AGHDASSI_Supplement1.pdf]

Supplementary material for "Surveillance of infections of surgical sites and lower respiratory tracts should be combined: experiences from the German surveillance module for operated patients (OP-KISS), 2018 to 2022"

Additional File 1

The German surveillance network KISS

The German national nosocomial infection system (Krankenhaus-Infektions-Surveillance-System, KISS) organised by the German National Reference Centre for Surveillance of Nosocomial Infections (NRC), serves as the primary HAI surveillance network in Germany. HAI surveillance for specific types of infection is mandatory in Germany (1). These include SSI, catheter-associated bacteraemia, ventilator-associated pneumonia, catheter-associated urinary tract infections, and *Clostridioides difficile* infections (2). Surveillance of non-ventilator associated pneumonia in general, or more specifically postoperative LRTI, is not mandatory. While HAI surveillance is obligatory, the methods of how hospitals conduct surveillance are not specified by law, and participation in KISS is voluntary. KISS is divided into several modules pertaining to different settings, patient populations and infections (3). Data for KISS are collected by local staff that must undergo methodological training organised by the NRC. Participants enter data into a designated online portal (webKess) provided and maintained by the NRC (https://webkess.charite.de/webkess2). Annually, the NRC hosts KISS participants' meetings to exchange experiences, present current data and trends, and introduce new elements.

Selected methodological aspects of OP-KISS

Participating departments choose one or several indicator procedures for which they conduct SSI surveillance. Besides a matching procedure code for the respective indicator procedure, prerequisites for including an operation in the OP-KISS surveillance, are primary wound closure in the operating theatre, and no prior surgery in 30 or 90 days (depending on the indicator procedure) before the procedure. OP-KISS follows a patient-based surveillance approach, meaning that surveillance does not end if a patient is transferred to another ward outside the surgical department. SSI surveillance ends after 30 or 90 days (depending on the indicator procedure), or prematurely in case of re-operation or death. LRTI surveillance ends after 30 days for all indicator procedures, or prematurely in case of re-operation, death, and unlike for SSI surveillance, at hospital discharge (4).

Key criteria of case definitions for pneumonia and bronchitis

For pneumonia, at least one, in patients with underlying cardiac or pulmonary disease at least two, radiological examination(s) suggestive of pneumonia, and one unspecific sign of infection (e.g. fever) had to present. Additionally, at least two specific symptoms (e.g. worsening gas exchange) had to be recorded. Alternately, if the causative microorganism had been detected, one specific symptom was sufficient.

Bronchitis was to be documented in cases, where radiological criteria for pneumonia were not fulfilled, but the causative microorganism had been detected in tracheal secretion or bronchoalveolar lavage. Additionally, two symptoms of infection had to be present.

References:

1. Kerwat K, Just M, Wulf H. [The German Protection against Infection Act (Infektionsschutzgesetz (IfSG))]. Anasthesiol Intensivmed Notfallmed Schmerzther. 2009;44(3):182-3.
2. Surveillance nosokomialer Infektionen sowie die Erfassung von Krankheitserregern mit speziellen Resistenzen und Multiresistenzen. Bundesgesundheitsblatt - Gesundheitsforschung - Gesundheitsschutz. 2013;56(4):580-3.
3. Schroder C, Schwab F, Behnke M, Breier AC, Maechler F, Piening B, et al. Epidemiology of healthcare associated infections in Germany: Nearly 20 years of surveillance. Int J Med Microbiol. 2015;305(7):799-806.
4. German National Reference Centre for Surveillance of Nosocomial Infections. [Surveillance of postoperative lower respiratory tract infections]. Berlin: NRZ; 2020. Available from: https://www.nrz-hygiene.de/files/Protokolle/OP-Protokolle/Atemweginfektionen/OP_KISS_Protokoll_AWI_v202011.pdf.

Disclaimer: This supplementary material is hosted by *Eurosurveillance* as supporting information alongside the article [Surveillance of infections of surgical sites and lower respiratory tracts should be combined: experiences from the German surveillance module for operated patients (OP-KISS), 2018 to 2022] on behalf of the authors who remain responsible for the accuracy and appropriateness of the content. The same standards for ethics, copyright, attributions and permissions as for the article apply. *Eurosurveillance* is not responsible for the maintenance of any links or email addresses provided therein.
