# Supplementary material for: Surveillance of infections of surgical sites and lower respiratory tracts should be combined: experiences from the German surveillance module for operated patients (OP-KISS), 2018 to 2022
Source: Euro Surveill. 2024 Mar 14;29(11):2300416. doi: 10.2807/1560-7917.ES.2024.29.11.2300416 (PMC10941308; doi:10.2807/1560-7917.ES.2024.29.11.2300416)
Supplement: Supplementary Material 2 [file 23-00416_AGHDASSI_Supplement2.pdf]

Supplementary material for "Surveillance of infections of surgical sites and lower respiratory tracts should be combined: experiences from the German surveillance module for operated patients (OP-KISS), 2018 to 2022"

Additional File 2

Table S1 Performance of lower respiratory tract infection (LRTI) surveillance by surgical departments participating in OP-KISS. Data from 2018 to 2022 per individual year

Year	Procedure type	Number of departments with LRTI surveillance (percentage among all OP-KISS departments)	Number of procedures with LRTI surveillance (percentage among all OP-KISS procedures)
2018	All procedures	25 (2.1)	4,112 (1.4)
	Lobectomy	9 (30)	951 (39.1)
	Abdominal surgery	3 (1.0)	267 (0.7)
	Traumatology and orthopaedics	8 (1.5)	1,710 (1.2)
2019	All procedures	25 (2.0)	4,376 (1.5)
	Lobectomy	10 (34.5)	1,030 (40.6)
	Abdominal surgery	5 (1.7)	500 (1.2)
	Traumatology and orthopaedics	7 (1.3)	2,428 (1.6)
2020	All procedures	24 (2.0)	3,639 (1.4)
	Lobectomy	8 (29.6)	838 (39.7)
	Abdominal surgery	7 (2.4)	543 (1.5)
	Traumatology and orthopaedics	8 (1.5)	2,085 (1.6)
2021	All procedures	33 (2.9)	5,672 (2.4)
	Lobectomy	7 (26.9)	601 (32.4)
	Abdominal surgery	5 (1.7)	234 (0.7)
	Traumatology and orthopaedics	14 (2.8)	1,238 (1.1)
2022	All procedures	26 (2.3)	5,440 (2.1)
	Lobectomy	7 (25.9)	580 (28.9)
	Abdominal surgery	5 (1.7)	128 (0.4)
	Traumatology and orthopaedics	8 (1.6)	1,404 (1.1)

Table S2 Surgical site infections (SSI), lower respiratory tract infections (LRTI) and combined postoperative infections per 100 operations. Data from 2018 to 2022 per individual year

Year	Procedure type	Number of procedures with SSI surveillance	SSI rate (per 100 operations)	Number of procedures with LRTI surveillance	LRTI rate (per 100 operations)	Combined postoperative infection rate (per 100 operations)
2018	Lobectomy	2,430	1.6	951	2.6	4.2
	Abdominal surgery	39,692	3.8	267	0	3.8
	Traumatology and orthopaedics	148,190	0.8	1,710	0.2	1.0
2019	Lobectomy	2,534	1.5	1,030	3.0	4.5
	Abdominal surgery	42,727	3.3	500	0.6	3.9
	Traumatology and orthopaedics	147,932	0.8	2,428	0.1	0.9
2020	Lobectomy	2,109	1.5	838	2.5	4.0
	Abdominal surgery	35,735	3.0	543	0.4	3.3
	Traumatology and orthopaedics	126,996	0.8	2,085	0	0.8
2021	Lobectomy	1,855	1.5	601	4.8	6.3
	Abdominal surgery	34,532	3.2	234	0	3.2
	Traumatology and orthopaedics	111,567	1.0	1,238	0.1	1.0
2022	Lobectomy	2,008	1.4	580	6.6	8.0
	Abdominal surgery	33,797	3.4	128	0.8	4.2
	Traumatology and orthopaedics	131,513	0.9	1,404	0	0.9

Disclaimer: This supplementary material is hosted by *Eurosurveillance* as supporting information alongside the article [Surveillance of infections of surgical sites and lower respiratory tracts should be combined: experiences from the German surveillance module for operated patients (OP-KISS), 2018 to 2022] on behalf of the authors who remain responsible for the accuracy and appropriateness of the content. The same standards for ethics, copyright, attributions and permissions as for the article apply. *Eurosurveillance* is not responsible for the maintenance of any links or email addresses provided therein.
